# A Review: Synthesis and Applications of Titanium Sub-Oxides

**DOI:** 10.3390/ma16216874

**Published:** 2023-10-26

**Authors:** Xiaoping Wu, Haibo Wang, Yu Wang

**Affiliations:** 1 State Key Laboratory of V and Ti Resources Comprehensive Utilization, Ansteel Research Institute of Vanadium & Titanium (Iron & Steele), Panzhihua 617000, China; 15273187604@163.com; 2 The School of Chemistry and Chemical Engineering, State Key Laboratory of Power Transmission Equipment & System Security and New Technology, Chongqing University, 174 Shazheng Street, Shapingba District, Chongqing 400044, China; wangy@cqu.edu.cn

**Keywords:** synthesis, application, titanium sub-oxides, Magnéli phase

## Abstract

Magnéli phase titanium oxides, also called titanium sub-oxides (Ti_n_O_2n−1_, 4 < n < 9), are a series of electrically conducting ceramic materials. The synthesis and applications of these materials have recently attracted tremendous attention because of their applications in a number of existing and emerging areas. Titanium sub-oxides are generally synthesized through the reduction of titanium dioxide using hydrogen, carbon, metals or metal hydrides as reduction agents. More recently, the synthesis of nanostructured titanium sub-oxides has been making progress through optimizing thermal reduction processes or using new titanium-containing precursors. Titanium sub-oxides have attractive properties such as electrical conductivity, corrosion resistance and optical properties. Titanium sub-oxides have played important roles in a number of areas such as conducting materials, fuel cells and organic degradation. Titanium sub-oxides also show promising applications in batteries, solar energy, coatings and electronic and optoelectronic devices. Titanium sub-oxides are expected to become more important materials in the future. In this review, the recent progress in the synthesis methods and applications of titanium sub-oxides in the existing and emerging areas are reviewed.

## 1. Introduction

Titanium sub-oxides, often referred to as Magnéli phase TiO_x_, comprise a series of different titanium oxides that have the general formula Ti_n_O_2n−1_ (4 ≤ n ≤ 10) [1,2,3,4,5]. It is well-known that titanium dioxide (TiO_2_) is an electrical insulator, as it has a large band gap (anatase: 3.2 eV; 3.0: rutile) [6]. However, Magnéli phase titanium oxides are electrically conducting, and the value of electrical resistivity decreases with the increase in the oxygen deficiency [7]. Furthermore, Magnéli phase TiO_x_ are found to be more stable than carbon in electrochemically oxidizing conditions [1,8]. These titanium sub-oxides have attracted much recent attention as promising new conducting materials because they are electrically conducting, and are highly stable towards chemical corrosion.

The earliest phase analysis using X-ray methods on the oxygen–titanium system was carried out by Ehrlich, who reported the existence of three intermediary titanium oxides [9,10]. In the 1950s, a phase diagram of a titanium–oxygen system was constructed from data in the literature by DeVries et al. [11]; later, a comprehensive phase analysis of TiO_x_ was studied by the group of Arne Magnéli, and a number of phases in the titanium–oxygen system were reported, including Ti_4_O_7_, Ti_5_O_9_, Ti_6_O_11_, Ti_7_O_13_, Ti_8_O_15_, Ti_9_O_17_ and Ti_10_O_19_ [12]. The electrical properties of these titanium oxides were studied by Bartholomew et al., and it was found that titanium sub-oxides have semiconductor-to-metal transitions at certain temperatures and their electrical conductivities change with the oxygen content of those materials [13]. Ti_4_O_7_ has the highest electrical conductivity among Magnéli phase TiO_x_ at room temperature [1].

Magnéli phase TiO_x_ are generally synthesized by the reduction of TiO_2_. The reduction sequence is as follows: TiO_2_ → Ti_n_O_2n−1_ (n > 10) → Ti_n_O_2n−1_ (4 < n < 10) → Ti_3_O_5_ → Ti_2_O_3_ → TiO → Ti_2_O [14]. Oxygen defect formations and its concentration depend on the synthesis conditions. Ti_4_O_7_ can be expressed as TiO_1.75_, as the ratio of O/Ti of Ti_4_O_7_ is 1.75. The Magnéli phase Ti_n_O_2n−1_ are the intermediate products. It is critical to have well-controlled preparation conditions to synthesize each Magnéli phase, with high chemical and phase purities that have significant effects on the intrinsic properties such as electrical, optical behavior and their catalytic activity.

It is increasingly important to synthesize nanostructured titanium sub-oxides because of their particular properties resulting from the high surface areas of the materials [15,16,17,18]. Progress has been made in applying nanostructured titanium sub-oxides in the areas of fuel cells, water treatment, batteries and so on. To update the recent research progress of synthesis and applications of titanium sub-oxides, this review will discuss and highlight the recent developments in the synthesis and applications of Ti_4_O_7_ in the fields of energy, environment, catalysts and others, as well as future directions for research.

## 2. Synthesis Methods

**Figure 1:**
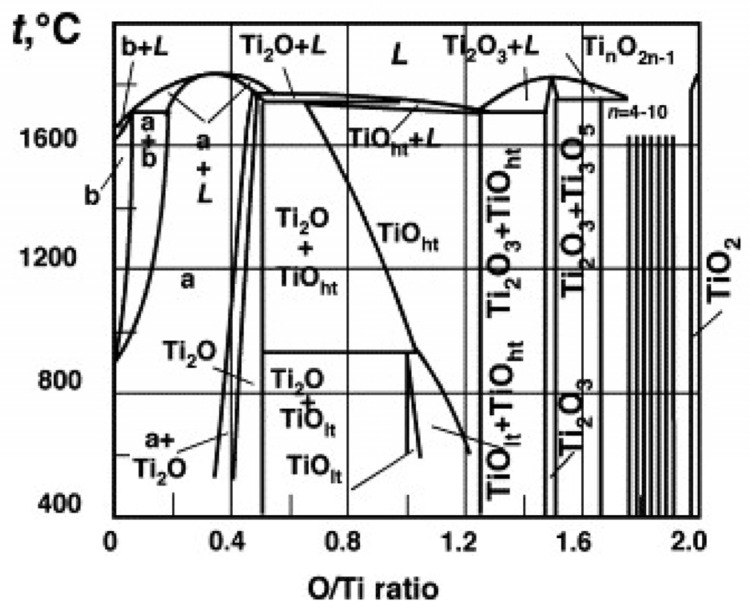
Phase diagram of the Ti–O system (Reprinted from ref. [14], copyright 2018, MDPI).

The phase diagram of the Ti–O system (Figure 1) shows various stable phases at different O/Ti ratios. The region at the right of the diagram contains the discrete Magnéli phases of Ti_n_O_2n−1_ (n = 4–10) and TiO_2_. At sufficiently elevated temperatures, TiO_2_ can be reduced to a lower oxidation state such as Magnéli series, including Ti_4_O_7_. To obtain individual phases such as Ti_4_O_7_ in the Magnéli series, the condition for reduction of TiO_2_ needs to be carefully controlled. The key parameters for the synthesis of Ti_4_O_7_, as well as for other Magnéli phases, include temperature, time, reducing atmosphere and reducing agents. Hydrogen, carbon, metal and hydride can be used as reducing agents.

### 2.1. Reduction of TiO_2_ by Hydrogen

At sufficiently elevated temperatures, hydrogen (H_2_) or a mixture hydrogen–inert gas such as argon (Ar) is used to reduce TiO_2_ into the titanium sub-oxides [1]. The reduction process can be considered to be a reaction of oxygen being removed progressively from TiO_2_. The reaction for the synthesis of Ti_4_O_7_ through hydrogen reduction is shown in Equation (1):

4TiO_2_ + H_2_ = Ti_4_O_7_ + H_2_O (1)

The reduction reaction to produce Ti_4_O_7_ is carried out at sufficiently elevated temperature, generally higher than 1000 °C. The sequence of the formation of Magnéli series TiO_x_ in the hydrogen reduction reactions of TiO_2_ is Ti_9_O_17_, Ti_8_O_15_, Ti_7_O_13_, Ti_6_O_11_, Ti_5_O_9_ and Ti_4_O_7_. The reduction reaction of TiO_2_ is carried out in a flow of hydrogen in a reactor that is heated externally to maintain a high temperature. As Ti_4_O_7_ is the last in the reduction sequence, the synthesis of Ti_4_O_7_ needs higher temperature, longer reduction time or a combination of two, compared with the parameters to the formation of other Magnéli series. The reaction temperature, reaction time, gas composition and size of TiO_2_ particles are important factors for the synthesis of Magnéli phases [2,3]. A summary of the synthesis of Ti_4_O_7_ through hydrogen reduction can be found in Table 1.

### 2.2. Reduction by Carbon

Titanium dioxide can be reduced by carbon in an inert atmosphere to produce various titanium sub-oxides, as shown in Equation (2):

nTiO_2_ (s) + C (s) = Ti_n_O_2n−1_ (s) + CO (g) (2)

The carbothermal reduction of TiO_2_ is a complex process in which the oxygen in TiO_2_ is progressively removed by carbon. TiO_2_ is initially reduced to Ti_n_O_2n−1_, Ti_3_O_5_, Ti_2_O_3_ and TiC_x_O_y_ [25,37,38,39], but an over stoichiometric carbon/TiO_2_ ratio may lead to the formation of TiC_x_O_y_, not titanium sub-oxides. Ti_n_O_2n−1_ phases are only formed as intermediates [25,37]. To prepare titanium sub-oxides through the carbothermal reduction of TiO_2_, the stoichiometric carbon/TiO_2_ ratio is important for the control of the phases formed. The carbothermal reduction of TiO_2_ can be carried out in different gas atmospheres or in a vacuum. Li et al. synthesized Ti_4_O_7_ by reacting TiO_2_ anatase (100 nm) with carbon black at 1020 °C for 0.5–2 h in argon and in a vacuum [26]. The study indicated that at the same temperature, the extent of carbothermal reduction of titanium dioxide is dependent on the molar ratio of TiO_2_/C, and excessive carbon may lead to over reduction down the sequence of titanium sub-oxides. Ti_4_O_7_ with a purity of 98.5% was obtained in argon at 1100 °C. Dewan et al. studied the carbothermal reduction of TiO_2_ in hydrogen, helium and argon through temperature-programmed reduction experiments [40]. In argon and helium, the carbothermal reduction of TiO_2_ started at 850 °C. In hydrogen, they found that the phases in a sample after being reduced to 915 °C were Ti_8_O_15_ and unreacted TiO_2_, and the phase in a sample after being reduced to 975 °C was only Ti_4_O_7_. Ti_4_O_7_ and Ti_3_O_5_ phases were found at 1035 °C.

Titanium sub-oxide fibers with high electrical conductivity have been prepared by reducing TiO_2_ in a carbon black micro-environment [29]. Organic polymers or compounds can be used to synthesize titanium sub-oxides. The carbon in the organic polymers is used as a carbon source for reducing TiO_2_ or other titanium-containing compounds. These organic polymers or compounds include poly (ethyleneimine), polyethyleneglycol [41], poly (styrene-b-2-vinylpyridine) [42], resol [43], glucose [44] and poly (vinyl alcohol) [27]. A summary of the various preparation methods for titanium sub-oxides using the carbon reduction method can be found in Table 1.

### 2.3. Reduction by Metals

Metals can be used to reduce TiO_2_ to form titanium sub-oxides [31,32]. Calcium, aluminum, sodium, silicon and titanium have been used to reduce TiO_2_. For example, by controlling the ratio of metallic titanium and TiO_2_, metallic titanium (Ti) can be used to reduce TiO_2_ to obtain various titanium sub-oxides through a reaction shown in Equation (3).

(2n−1)TiO_2_(s) + Ti(s) = 2Ti_n_O_2n−1_(s) (3)

Andersson et al. synthesized various titanium sub-oxides by the reduction of TiO_2_ with titanium metal under argon, and established the different phases from X-ray diffraction determinations [9]. Strobel et al. used Ti and TiO_2_ to react in situ in carefully out-gassed transport tubes. Cl_2_ and tellurium tetrachloride were used as transporting agents to synthesize crystals of Ti_n_O_2n−1_ with n = 2 to 9 [45]. Gusev et al. developed a method for the synthesis of titanium sub-oxides by reducing TiO_2_ with titanium. This method involved the mechanical activation and annealing in argon at temperatures of 1333–1353 K for 4 h [46]. It is worth noting that the synthesis of titanium sub-oxides using TiO_2_ and Ti can be viewed to be an oxidation reaction in which Ti is oxidized by TiO_2_. Theoretically, oxidation of Ti is one of the possible ways to obtain titanium suboxides. However, oxygen is highly reactive and can oxidize Ti directly to TiO_2_ easily. Fine Ti powder is far more difficult to prepare than TiO_2_. A summary of the synthesis of titanium sub-oxides by metal reduction can be found in Table 1.

### 2.4. Reduction by Hydride

Metal hydrides have strong reducing reactivity, even at low temperatures. The reduction of TiO_2_ to titanium sub-oxides could occur at low temperatures to avoid significant sintering and crystal growth of particles in the formation process of titanium sub-oxides. Therefore, metal hydrides could be used to synthesize nanostructured titanium sub-oxides using nanostructured TiO_2_ as a starting material. Nagao et al. synthesized titanium sub-oxides by reacting TiO_2_ with TiH_2_ at 550 °C [34]. The nanoparticles of a series of phases of titanium sub-oxide including Ti_2_O_3_, Ti_3_O_5_, Ti_4_O_7_ and Ti_8_O_15_ were obtained by changing the molar ratios of TiO_2_/TiH_2_. Other hydride reduction methods for titanium sub-oxide synthesis can be found in Table 1.

### 2.5. Synthesis of Nanostructured Titanium Sub-Oxides

It has become increasingly important to synthesize nanostructured titanium sub-oxides because of their particular properties resulting from the high surface areas of the materials. Nanostructured non-stoichiometric TiO_2−x_ titanium sub-oxides, titanium sub-oxides Ti_n_O_2n−1_ in particular, have emerged as alternatives to TiO_2_ in applications of clean energy generation, and as catalysts for degrading harmful compounds and others [47,48,49,50,51,52]. Although titanium sub-oxides can be synthesized by the reduction of TiO_2_ using hydrogen or carbon, the sizes of synthesized titanium sub-oxide particles are usually in the order of micrometers, because these reduction reactions occur at high temperatures (generally over 1000 °C) and proceed for hours. Under these conditions, TiO_2_ and formed titanium sub-oxide particles undergo sintering and crystal growth, leading to the formation of much larger particles. To synthesize nanostructured titanium sub-oxides, more reactive titanium-containing starting materials, stronger reducing agents or alternative reaction techniques are required for the reduction reactions to be carried out under milder reaction conditions such as lower temperatures or short reaction times.

**Figure 2:**
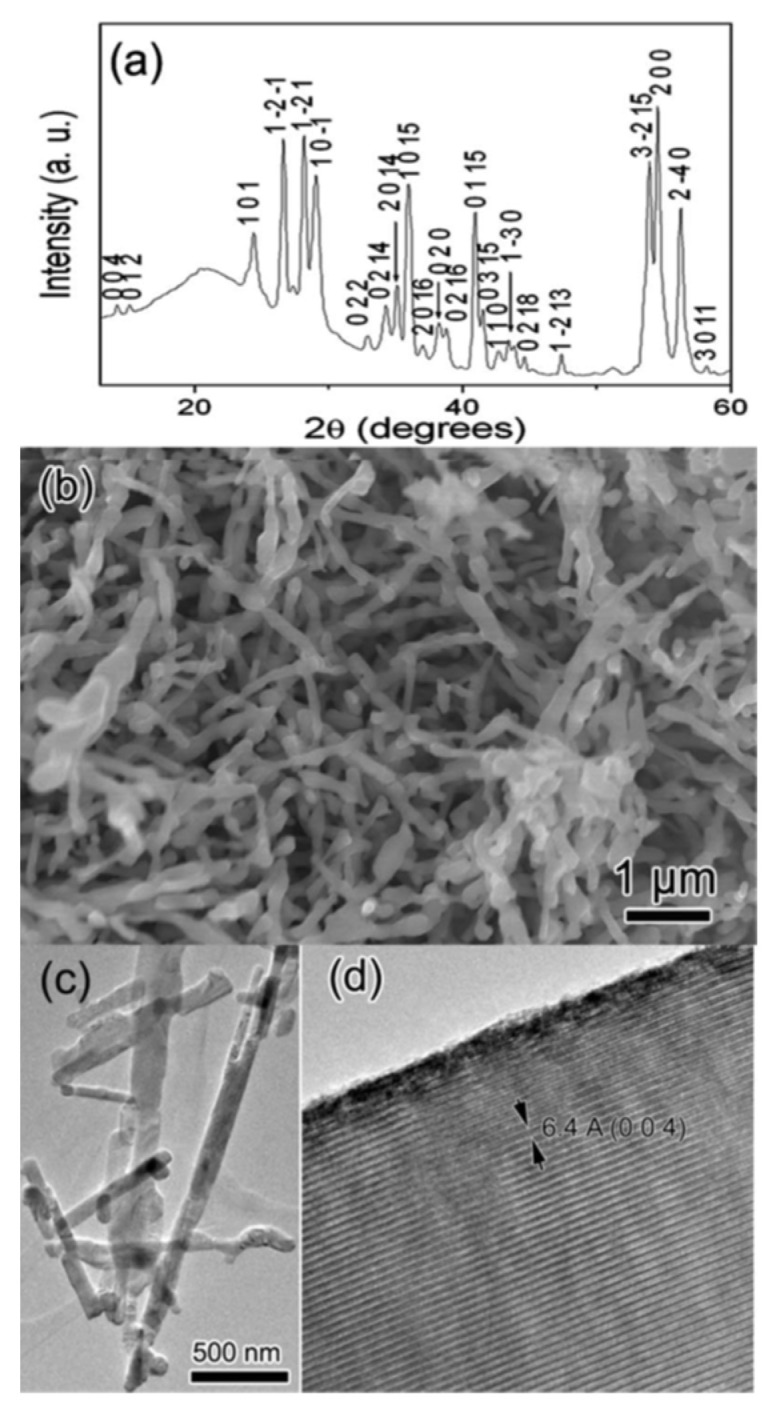
Ti_8_O_15_ nanorods prepared by reducing H_2_Ti_3_O_7_ at 850 °C in hydrogen; (**a**) XRD pattern of the product; (**b**) SEM image of the product; (**c**) low magnification TEM image of the product; and (**d**) a high-magnification TEM image of part of a nanorod (Reprinted from ref. [53], copyright 2008, American Institute of Physics).

Han et al. prepared Ti_8_O_15_ nanowires and Ti_4_O_7_ fibers by heating H_2_Ti_3_O_7_ nanowires in hydrogen at 850 °C and 1050 °C [53]. Hydrogen trititanate H_2_Ti_3_O_7_ is one of the compounds in the series of titanates (M_2_Ti_n_O_2n+1_, M = H, Na, or K). The synthesis process has two steps. Firstly, H_2_Ti_3_O_7_ nanowires are prepared by reacting TiO_2_ particles with NaOH in an autoclave at 150–180 °C for 2–5 days, and then purified using the acid washing method [54,55,56]. Scanning electron microscopy (SEM) and transmission electron microscopy (TEM) studies showed that prepared H_2_Ti_3_O_7_ were nanowires of 30–200 nm in diameter and up to 10 µm long. Secondly, prepared H_2_Ti_3_O_7_ nanowires were reduced in hydrogen for 1–4 h at 800–1050 °C. In the hydrogen reduction reaction at 850 °C, H_2_Ti_3_O_7_ nanowires changed into Ti_8_O_15_ nanorods or nanoparticles, as shown in Figure 2. By heating H_2_Ti_3_O_7_ in hydrogen at 1050 °C, the product formed was Ti_4_O_7_. The TEM image shows that most products are in the form of fibers, with diameters of approximately 1 µm.

**Figure 3:**
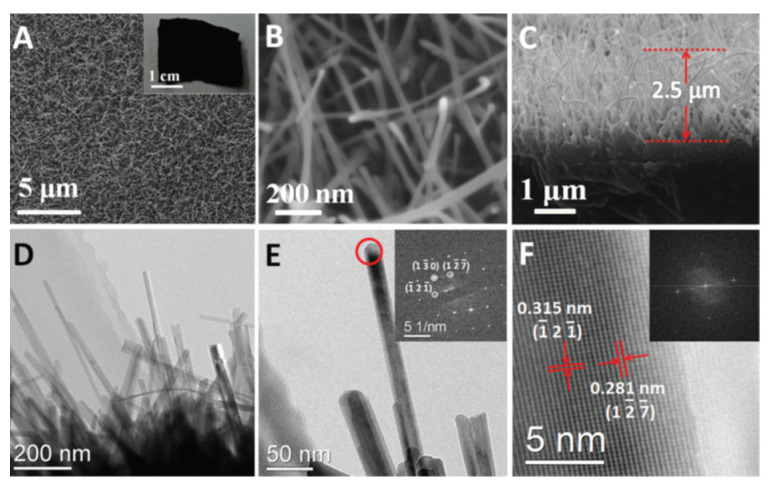
(**A**,**B**) SEM images of the Ti_8_O_15_ nanowires; (**C**) SEM image of the cross-section of the Ti_8_O_15_ nanowires; (**D**,**E**) TEM images of the Ti_8_O_15_ nanowires; (**F**) HRTEM image of Ti_8_O_15_ nanowires (Reprinted from ref. [15], copyright 2015, The Royal Society of Chemistry).

He et al. fabricated Ti_8_O_15_ nanowires using an evaporation–deposition synthesis method [15]. The synthesized Ti_8_O_15_ nanowires were ∼30 nm in diameter (as shown in Figure 3), and were found to have an electrical conductivity of 20.6 S cm^−1^.

Zhang et al. prepared pure Ti_4_O_7_ particles with diameters of 200–500 nm in hydrogen at 850 °C using peroxotitanium acid H_4_TiO_5_ (Ti(OH)_3_O-O-H) as a starting material [57]. H_4_TiO_5_ was prepared by treating titanium powder with NH_3_·H_2_O and H_2_O_2_. Pang et al. synthesized Ti_4_O_7_, using a simple polymer-mediated route in which the cross-linked titanium ethoxide with polyethylene glycol was treated by carbothermal reduction at ~950 °C in an Ar stream. TEM images revealed that the material primarily comprises ~8–20 nm Ti_4_O_7_ crystals. The sulfur composites Ti_4_O_7_/S-60 or Ti_4_O_7_/S-70 were prepared with either 60 or 70 wt% sulfur using a melt-diffusion method at 155 °C [58]. Ti_4_O_7_ was used to prepare Ti_4_O_7_/S cathodes for lithium–sulfur cells [59,60,61].

**Figure 4:**
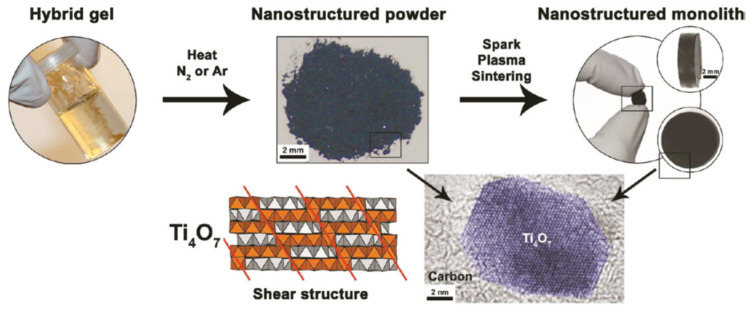
Synthesis steps for Magnéli/carbon nanocomposites (Reprinted from ref. [41], copyright 2011, American Chemical Society).

Portehault et al. developed a new bottom-up approach to synthesize various nano-scaled Magnéli phases under mild conditions [41]. In this method, titanium (IV) ethoxide was reacted with amino- or ethoxy-containing oligomers or polymers. The resulting clear gels were heated at different temperatures under N_2_ or Ar. Ti_n_O_2n−1_ compounds (n = 3, 4, 5, 6, 8) were obtained for the first time as nano-Magnéli phases with specific surface areas from 55 to 300 m^2^ g^−1^. The synthesis steps for the Magnéli/carbon nanocomposites are illustrated in Figure 4.

Huang et al. synthesized nanocrystalline Ti_2_O_3_, Ti_3_O_5_ and Ti_4_O_7_ using a synthesis method that combines sol-gel and vacuum-carbothermic processes [44]. Yao et al. successfully synthesized Ti_4_O_7_ using TiO(NO_3_)_2_ as a starting material in a hydrogen atmosphere at 1000 °C for 6 h [22]. The SEM images clearly showed that the synthesized titanium sub-oxides are spherical particles with an average particle size of approximately 250 nm. Davydov synthesized Ti_4_O_7_ nanopowder with an average size of 115 ± 30 nm using a two-step procedure. In the first step, titanium (III) oxalate particles with controlled sizes were produced by reacting metallic Ti with oxalic acid in a heated aqueous solution. In the second step, Ti_4_O_7_ was prepared through high-temperature calcination of titanium (III) oxalate particles in a flowing hydrogen gas [62]. This synthesis process is similar to the process that has been used to prepare titanium oxycarbide nanoparticles [63]. Tominaka et al. synthesized Ti_2_O_3_ nanoparticles by heating TiO_2_ nanoparticles (10–30 nm) and CaH_2_ powder at 350 °C [35].

Ioroi et al. synthesized nanoparticles of titanium sub-oxides by irradiating TiO_2_ particles dispersed in liquid with a pulsed UV laser [64]. Xu et al. developed a synthesis process to prepare titanium sub-oxide nanoparticles via a thermal plasma method, using metatitanic acid H_2_TiO_3_ (TiO(OH)_2_) as a starting material. The prepared titanium sub-oxides nanoparticles are spherical, with particle sizes in the range of 20–100 nm [23]. Fukushima et al. synthesized Ti_4_O_7_ nanoparticles with different sizes by carbothermal reduction using a multimode microwave apparatus [28]. Takeuchi et al. synthesized 60 nm Ti_4_O_7_ nanoparticles via carbothermal reduction of TiO_2_ nanoparticles using polyvinylpyrrolidone as the carbon source. The carbothermal reduction was carried out using 2.45 GHz microwave irradiation at 950 °C for 30 min. The results of this study demonstrate that microwave heating can drastically reduce the heating time to avoid excessive sintering and crystal growth of Ti_4_O_7_ in a conventional carbothermal reduction process [65]. Arif et al. prepared chain-structured titanium sub-oxides with diameters under 30 nm using a thermal-induced plasma process. The synthesized titanium sub-oxide nanoparticles consisted of a mixture of several Magnéli phases. After a heat treatment, as-synthesized titanium sub-oxides nanoparticles were found to have low electrical resistivity [66].

A summary of synthetic methods for nanostructured titanium sub-oxides is reported in Table 2. A comparison among the synthesis methods to highlight the advantages, limitations and characteristics of the prepared sub-oxides is presented in Table 3.

## 3. Applications of Titanium Sub-Oxides

**Figure 5:**
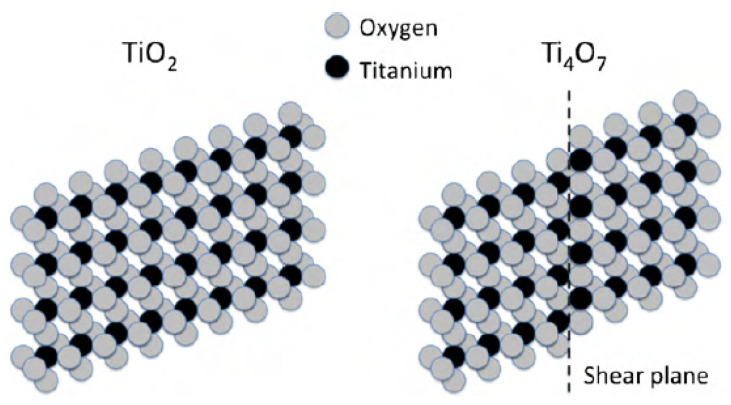
Edge-sharing TiO_2_ and Ti_4_O_7_ octahedra sheets showing the face-sharing shear plane in Ti_4_O_7_ (Reprinted from ref. [68], copyright 2010, Elsevier).

The structures of Magnéli phase titanium oxides are based on the rutile TiO_2_ crystal lattice. Rutile TiO_2_ is made up of octahedra having a titanium atom in the center and oxygen atoms at each corner. Shared edge or corner oxygen atoms link adjacent octahedra, as shown in Figure 5. The crystal structure of titanium sub-oxides can be described as a structure having a two-dimensional chain of titanium dioxide in which titanium atoms locate at the center and oxygen atoms locate at the corners in an octahedral structure [67,68]. In Ti_n_O_2n−1_, every nth layer has an oxygen deficiency, which leads to shear planes in the crystal structure. The Ti_4_O_7_ crystal has three octahedral TiO_2_ layers and one TiO layer. As a result of the vacancy of oxygen atoms, the TiO layer causes titanium atoms to be closer together.

The unique crystal structure makes titanium sub-oxide materials have attractive properties, such as high conductivity, superior chemical stability and electrochemical stability [69]. As shown in Table 4, the conductivity of Ti_4_O_7_ material is the highest among the Magnéli phase materials. Research shows that Ti_4_O_7_ is highly stable in acidic or alkali conditions. Some studies indicated that the expected half-life of Ti_4_O_7_ is 50 years in 1.0 M H_2_SO_4_ at room temperature [70].

As a result of their remarkable electrical conductivity, electrochemical stability, cost-effectiveness and environmentally friendly natures, titanium sub-oxides are also considered to have potential as a superior anode material for wider electrochemical applications [67,68]. Research has shown that Magnéli phases have a catalytic property. Among the Magnéli phases, Ti_4_O_7_ exhibits the greatest catalytic property [45,69]. It has a wide electrochemical window with regard to water oxidation and reduction [70,71,72]; thus, it can be used for electrochemical treatment of pollutants in water. Titanium sub-oxides are generally prepared in the form of powders. More recently, two-dimensional films and three-dimensional porous materials of titanium sub-oxides have been successfully fabricated. Advances in the development of multiple dimensional titanium sub-oxide materials has led to new applications.

### 3.1. Catalysis Support in Fuel Cells

Proton exchange membrane fuel cells (PEMFCs) are a clean energy technology that has made significant advances in recent decades [73,74]. However, the high cost of the component materials and the low stability of the electrodes are major barriers for their large-scale commercial applications in some areas [75]. PEMFCs use Pt catalysts in the form of nanoparticles dispersed on a support material. The nature of the support materials can have a significant influence on the electro-activity and durability of the Pt catalysts [76,77,78]. Carbon materials are the most common support material for PEMFCs. However, carbon-supported Pt catalysts are prone to corrosion under the harsh operating conditions [79,80], which can severely affect the performance of PEMFCs and reduce the operational lifetime of the fuel cell electrodes. Titanium sub-oxides are considered to be promising support materials for PEMFCs due to the high thermal and oxidative stability, electronic conductivity and strong interactions between Pt nanoparticles and titanium sub-oxide support [81]. Chisaka et al. synthesized Ti_4_O_7_ particles via carbothermal reduction, using titanium oxysulfate (TiOSO_4_) and polyethylene glycol as precursors. The Pt catalyst using Ti_4_O_7_ as support exhibited excellent load cycle durability, which was the highest among the state-of-the-art platinum/oxide catalysts, with no change in the cell performance after 10,000 voltage cycles [82]. Esfahani et al. synthesized doped titanium sub-oxide Ti_3_O_5_Mo_0.2_Si_0.4_ (TOMS) as a novel fuel cell catalyst support. Ti_3_O_5_Mo_0.2_Si_0.4_ (TOMS) support exhibited remarkably high electronic conductivity and high stability. The fuel cell devices that used the Pt/TOMS catalyst achieved high performance, better than that of commercial catalysts [83]. Nguyen et al. demonstrated the excellent durability of titanium sub-oxide as a catalyst support for Pd in alkaline direct ethanol fuel cells [84]. Won et al. developed Ti_4_O_7_-supported Pt-based catalysts for a bifunctional oxygen catalyst in a unitized regenerative fuel cell for both the oxygen reduction reaction (ORR) and the oxygen evolution reaction (OER) to enhance their activity and stability [85]. Zhang et al. developed an ordered Ag@Pd alloy supported on Ti_4_O_7_. The ordered characteristics of the Ag@Pd alloy and its strong electron transfer with the corrosion-resistant Ti_4_O_7_ improved the catalytic activity and stability [86].

### 3.2. Electrocatalytic Degradation for Wastewater Treatment

Titanium sub-oxides have been characterized as an ideal choice of anode for the electrochemical treatment of many pollutants. Chen et al. decomposed trichloroethylene (TCE) and chloroform (CF) in an electrochemical cell using a titanium sub-oxide ceramic sheet plated with Pt or Pd as the working electrode. The decomposition kinetics was found to be of the first order for TCE and CF [87]. Kearney et al. used Ebonex (a titanium sub-oxide ceramic) electrodes for treating nitrate-contaminated water. Complete de-nitrification was achieved using an Ebonex cathode and a stable anode based on Ti/IrO_2_ or Ti/RuO_2_ [88]. Yang et al. examined the degradation of perfluorooctanesulfonate in electrochemical oxidation processes, using an anode made from Ti_4_O_7_. The decomposition rate of perfluorooctanesulfonate was shown to be pseudo-first-order. This study illustrates the promise of Ti_4_O_7_ electrodes for degrading per- and polyfluoroalkyl compounds and co-contaminants in groundwater [89]. Ganiyu et al. reported a study of the electrochemical degradation of the antibiotic amoxicillin in aqueous solution. The Ti_4_O_7_ anode of the cell was prepared using plasma spraying technology. The oxidative degradation of amoxicillin by hydroxyl radicals was assessed as a function of the applied current, and was found to follow pseudo-first-order kinetics. Comparative studies of mineralization efficiency showed that a Ti_4_O_7_ anode performed better for the removal of total organic carbon (TOC) than the classical dimensional stable anode and Pt anode. Ti_4_O_7_ anodes could provide a cost-effective alternative to boron doped diamond anodes in electro-oxidation processes [90]. Teng et al. investigated the electrochemical oxidation of sulfadiazine using a Ti/Ti_4_O_7_ mesh anode. Their results showed that electrochemical oxidation could achieve almost 100% removal of sulfadiazine in 60 min under the conditions of 0.05 mol L^−1^ Na_2_SO_4_, pH = 6.33 and current density of 10 mA cm^−2^. It was found that Ti/Ti_4_O_7_ mesh anodes were very stable in the treatment of actual pharmaceutical wastewater, and had a large electrochemically active surface area due to the network structure of the Ti/Ti_4_O_7_ mesh anode [91]. Further research will continue to improve the performance of titanium sub-oxide electrodes through optimizing the fabrication process of the electrodes and further integrating them with other technologies for more efficient applications.

### 3.3. Reactive Electrochemical Membrane

One recent research advancement in water treatment concerns the development of technologies that incorporate multiple treatment methods into a single technology to increase the efficiency and reduce the complexity of water treatment. A novel technology known as reactive electrochemical membranes (REM) combines membrane filtration with electrochemically advanced oxidation processes. In this REM technology, titanium sub-oxide materials serve as both a ceramic membrane for filtration and a reactive electrode surface for oxidizing contaminants [92]. Zaky et al. used Ti_4_O_7_ REM to investigate the removal of p-substituted phenolic compounds in water. They demonstrated that the REM was active for both direct anodic oxidation and production of OH• radicals to degrade phenolic compounds [93]. Guo et al. synthesized a novel REMs for water treatment using tubular asymmetric TiO_2_ ultrafiltration membranes as precursors. REMs composed of high purity Ti_4_O_7_ showed optimal reactivity. The performance of REMs was assessed by measuring the outer-sphere charge transfer (Fe(CN)6^4−^) and oxidation of organic compounds through both direct oxidation and generation of OH•. In an optimal condition, the removal rate for oxalic acid was determined to be 401.5 ± 18.1 mmol h^−1^ m^−2^ at 793 L m^−2^ h^−1^. The current efficiency was approximately 84%. These results show the high promise of REMs in applications of water treatment [94]. Qi et al. prepared Ti_4_O_7_ REM by thermal reduction of mechanically pressed TiO_2_ powders, using the Ti powder as the reducing agent. The prepared Ti_4_O_7_ REMs show high oxygen evolution potential and electrocatalytic activity for the generation of OH• [95]. You et al. fabricated a monolithic porous Ti_4_O_7_ electrode for electrochemical oxidation of industrial dyeing and finishing wastewater. The electrochemical oxidation using porous Ti_4_O_7_ electrode produced efficient and stable reduction of recalcitrant organic pollutants onsite, without any extra addition of chemicals [96]. Geng et al. fabricated tubular Ti_4_O_7_/Al_2_O_3_ composite microfiltration membranes for electrically-assisted antifouling filtrations. The tubular Ti_4_O_7_/Al_2_O_3_ membrane was tested for its antifouling performance by treating different feed solutions that are known to foul easily in an electrically-assisted membrane filtration module. The results demonstrated that the Ti_4_O_7_/Al_2_O_3_ composite membranes showed much better antifouling performance than uncoated Al_2_O_3_ membranes. The incorporation of a Ti_4_O_7_-modified membrane into the electrically-assisted filtration process provides a potential alternative for ceramic membrane filtrations to have antifouling properties for maintaining long-lasting permeate quality and simplifying the filtration operation [97]. Liang et al. developed a REM system using a Ti_4_O_7_ microfiltration membrane as the filter and the anode. The REM system was evaluated for the performance in deactivating *Escherichia coli* (*E. coli*) in water at various current densities. The results showed that the concentration of *E. coli* was reduced from 6.46 log CFU/mL to 0.18 log CFU/mL, after passing through the Ti_4_O_7_ microfiltration membrane filter. The scanning electron microscope and extracellular protein analysis showed that the membrane filtration effect and direct oxidation generated from the REM system are responsible for the observed bacteria removal and inactivation [98]. Research is continuing to optimize the electrode fabrication process, and to develop titanium sub-oxide electrodes doped with active electrode materials to further increase the efficiency of water treatment processes, prolonging electrode working life and expanding the degradation of complex pollutants.

### 3.4. Batteries

The lithium–sulfur battery (LSB) is considered to be one of the next-generation technologies for future batteries because of its remarkable specific capacity of 1675 mA h g^−1^ and the availability of low-cost sulfur [60,99]. However, the development of commercial LSBs needs to resolve the issues of low sulfur utilization and poor cyclability, which are caused by a number of factors, such as the low conductivity of sulfur, the high solubility of the lithium polysulfides, passivation of the reactive surface of lithium anodes, and so on. To address these issues, one of the research efforts is to develop host materials to limit the movement of the lithium polysulfides in the sulfur cathode. Tao et al. discovered that conductive Ti_4_O_7_ was a highly effective matrix to bind with sulfur species. Ti_4_O_7_–S cathodes exhibit higher reversible capacity and improve cycling performance over previously developed TiO_2_–S cathodes. The strong adsorption of sulfur species on the low-coordinated Ti sites of Ti_4_O_7_ was attributed to the improved performance of Ti_4_O_7_–S cathodes [100]. Wei et al. prepared mesoporous Ti_4_O_7_ microspheres that exhibit interconnected mesopores (20.4 nm), large pore volume (0.39 cm^3^ g^−1^), and a high surface area (197.2 m^2^ g^−1^). The sulfur cathode embedded with a matrix of mesoporous Ti_4_O_7_ microspheres exhibits a superior reversible capacity and a low decay in capacity. The improved electrochemical performance is due to the strong chemical bonding of the lithium polysulfides to Ti_4_O_7_, and trapping in the mesopores and voids of the matrix [43]. Zhang et al. reported a facile approach to prepare nanostructured Ti_4_O_7_ with different morphologies. Ti_4_O_7_ nanorods and nanoparticles were prepared. The as-prepared Ti_4_O_7_ nanorods and nanoparticles were examined as a sulfur host for Li–S batteries. The electrochemical tests showed that the Ti_4_O_7_ nanorods exhibited better performance in cycle stability and rate capacity compared with Ti_4_O_7_ nanoparticles. This confirmed that the morphology of Ti_4_O_7_ could influence its electrochemical performance for lithium sulfur batteries [101]. Wu et al. synthesized a composite containing carbon nanotubes and nanosized Ti_4_O_7_ (oCNTs-Ti_4_O_7_), and coated the composite on the surface of the separator. Compared with a common separator, the separator modified with the oCNTs-Ti_4_O_7_ layer exhibited significantly improvement in the utilization of active substances, and restrained the shuttling effect of polysulfides. The Li–S battery fabricated using the separator modified with the oCNTs-Ti_4_O_7_ layer showed great enhancements in cycle and rate performance, as well as other in electrochemical properties [102]. Yu et al. fabricated a lithium–sulfur battery cathode containing 7.5 wt% to 10 wt% Ti_4_O_7_. The addition of Ti_4_O_7_ as a conductive additive into the cathode resulted in better rate capability and reversible cycling performance. The high electronic conductivity and surface adsorption of the polysulfides of Ti_4_O_7_ were attributed to the improvement in the electrochemical performance. This research also showed an effective way to improve the performance of lithium–sulfur batteries [103]. Titanium sub-oxides have also been used to improve the performance of other types of batteries such as lead–acid batteries and Zn–air batteries [104,105].

### 3.5. Other Applications

Titanium sub-oxides are also considered to be attractive for solar cells [106,107,108,109,110], sensors [111,112,113,114,115], electronic and photonic materials [3,7,16,116], and biological applications [117]. A summary of application areas of these materials in existing and emerging areas of research is listed in Table 5.

## 4. Summary and Outlook

In this review, recent progress in the synthesis and applications of Magnéli phase titanium oxides was reviewed. Titanium sub-oxides are synthesized through the reduction of titanium dioxide (TiO_2_) using hydrogen, carbon, metals or metal hydrides as reduction agents. The particle sizes of as-synthesized titanium sub-oxides are generally in the micrometer range, based on the conventional synthesis methods. However, progress has been made to synthesize nanostructured titanium sub-oxides through optimizing thermal reduction processes, using more powerful reduction agents or using new titanium-containing precursors [15,23,28,62,65,66]. Magnéli phase titanium oxides have numerous applications in electrodes, fuel cells, degradation of pollutants, batteries and coatings. Among these compounds, Ti_4_O_7_ has received the most widespread attention due to its excellent electrical conductivity, and chemical and electrochemical stability. More recently, Magnéli phase titanium oxides as functional materials or additives have been used to enhance the performance of electro-catalysts, cathodes in batteries, advanced electrochemical oxidation processes, solar cells, electronic materials, sensors and coatings [95,110,111,118,119,120,121,122]. It is expected that further research will be continue to optimize synthesis processes of Magnéli phase titanium oxides to further increase the electrochemical and catalytic properties, and to improve the performance of devices containing Magnéli phase titanium oxides through optimizing the fabrication process and further integrating with other technologies for more efficient applications. Titanium sub-oxides are expected to become more important materials for sustainability in the future.

## Figures and Tables

**Table 1:** Summary of synthesis of Ti_4_O_7_ and other titanium sub-oxides.

Synthesis Method	Process Conditions	Characterization	Ref.
H_2_ reduction	Pigmentary TiO_2_ reacts with H_2_ at 1050 °C	Monophasic Ti_4_O_7_	[4]
H_2_ reduction	Anatase TiO_2_ reacts with H_2_ (99.99%) at 950 °C	Pure triclinic phase of Ti_4_O_7_, 0.5–1 µm	[2]
H_2_ reduction	TiO_2_ nanotube arrays react with H_2_ at 850 °C for two hours	Ti_4_O_7_ nanotube arrays	[19]
H_2_ reduction	TiO_2_ reacts with H_2_	70% Ti_4_O_7_, 30% Ti_5_O_9_	[20]
H_2_ reduction	Rutile TiO_2_ was reduced in a mixture of N_2_ and H_2_ gases	Ti_4_O_7_, Ti_5_O_9_ and Ti_6_O_11_	[21]
H_2_ reduction	TiO(NO_3_)_2_ reacts with H_2_ at 1000 °C for 6 h	Ti_4_O_7_, 250 nm	[22]
H_2_ reduction	H_2_TiO_3_ reacts with H_2_/Ar in a thermal plasma reactor	Ti_n_O_2n−1_ nanoparticles, 20–100 nm	[23]
H_2_ reduction	TiO_2_ + H_2_ in a combined catalytic and thermal reduction reaction	Ti_8_O_15_, Ti_4_O_7_, Ti_3_O_5_	[24]
C reduction	The reduction of TiO_2_ by graphite or metallic titanium	Various phases	[25]
C reduction	TiO_2_ anatase (100 nm) reacts with carbon black at 1020 °C for 0.5–2 h	Ti_4_O_7_, 98.5%	[26]
C reduction	TiO_2_ reacts with poly(vinyl alcohol) at 1100 °C	Ti_4_O_7_, a few hundreds of nm in size	[27]
C reduction	TiO_2_ reacts with polymer PVP at 925 °C in a microwave furnace	Ti_4_O_7_ nanoparticles (25, 60, and 125 nm)	[28]
C reduction	Reduction of rutile TiO_2_ in a carbon black micro-environment	Titanium sub-oxide fibers	[29]
Metal reduction	Heating Ti and TiO_2_ in an electric arc furnace	Titanium sub-oxides	[12]
Metal reduction	Heating TiO_2_ and Ti metal in an evacuated silica tube at 1150 °C	Ti_4_O_7_ crystals	[30]
Metal reduction	Heating Ti and TiO_2_ in H_2_	Ti_4_O_7_	[31]
Metal reduction	Heating TiO_2_ and silicon powder or silicon/CaCl_2_ powder	Various titanium sub-oxide powders	[32]
Metal reduction	Reducing macroporous anatase TiO_2_ using a zirconium getter	Ti_n_O_2n−1_ (n = 2, 3, 4, 6)	[33]
Hydride reduction	Solid-phase reaction of TiO_2_ with TiH_2_ at relatively low temperature	Titanium sub-oxide nanoparticles	[34]
Hydride reduction	Heating TiO_2_ nanoparticles and CaH_2_ powder at 350 °C	Ti_2_O_3_ nanoparticles	[35]
Hydride reduction	TiO_2_ was embedded with CaH_2_ and heated at 360 to 500 °C.	TiO_x_ thin films	[36]

**Table 2:** A summary of methods for synthesis of nanostructured titanium sub-oxides.

Materials	Method	Ref.
Ti_8_O_15_ nanowires	Heating H_2_Ti_3_O_7_ nanowires in hydrogen at 850 °C	[53]
Ti_8_O_15_ nanowires	An evaporation–deposition synthesis method	[15]
Ti_4_O_7_ particles with diameters of 200–500 nm	Reduction of H_4_TiO_5_ with hydrogen at 850 °C	[57]
Ti_4_O_7_ crystals (8–20 nm)	Carbothermal reduction of cross-linked titanium ethoxide with polyethylene glycol at ~950 °C in Ar stream	[58]
Magnéli phases with specific surface areas from 55 to 300 m^2^ g^−1^	The gels made from titanium (IV) ethoxide and amino- or ethoxy-containing oligomers or polymers were heated at different temperatures under N_2_ or Ar	[41]
Nanocrystalline Ti_2_O_3_, Ti_3_O_5_ and Ti_4_O_7_	A combined sol-gel and vacuum-carbothermic processes	[44]
Ti_4_O_7_ particles (around 250 nm)	Reduction of TiO(NO_3_)_2_ in hydrogen at 1000 °C for 6 h	[22]
Ti_4_O_7_ nanopowder (115 ± 30 nm)	Reduction of titanium (III) oxalate particles in hydrogen	[62]
Ti_2_O_3_ nanoparticles	Heating TiO_2_ nanoparticles (10–30 nm) and CaH_2_ powder at 350 °C	[35]
Titanium sub-oxide nanoparticles	Irradiation of TiO_2_ particles dispersed in liquid with a pulsed UV laser	[64]
Titanium sub-oxide nanoparticles (20–100 nm)	By a thermal plasma method, using metatitanic acid (H_2_TiO_3_) as a starting material	[23]
Ti_4_O_7_ nanoparticles	Carbothermal reduction using a multimode microwave apparatus	[28]
Ti_4_O_7_ nanoparticles (60 nm)	Carbothermal reduction of TiO_2_ nanoparticles using microwave irradiation at 950 °C for 30 min	[65]
Titanium sub-oxides (30 nm)	A thermal-induced plasma process	[66]

**Table 3:** A comparative table among the synthesis methods.

Synthesis Methods	Advantages/Limitations	Characteristics
Hydrogen reduction	A simple, well established/handling reactive gas	For synthesis of multi-dimensional pure titanium sub-oxides
Carbon reduction	Use of various of carbon sources/uniform mixing of the reactants	For synthesis of various titanium sub-oxides by controlling mole ratio of carbon and TiO_2_
Metal reduction	Without handling reactive gas/controlling reaction process	Usually a mixture of different titanium sub-oxides
Hydride reduction	Reaction at relatively low temperature/handling reactive starting reactant	For synthesis of titanium sub-oxides with smaller particle sizes

**Table 4:** Electrical conductivity for single Magnéli phase materials *.

Ti_n_O_2n−1_ Phase	Electrical Conductivity (σ/S cm^−1^)	Log10 (σ/S cm^−1^)
Ti_4_O_7_	1995	3.3
Ti_5_O_9_	631	2.8
Ti_6_O_11_	63	1.8
Ti_8_O_15_	25	1.4

* Adopted from ref. [68], copyright 2010, Elsevier.

**Table 5:** Summary of application areas of Magnéli phases.

Area	Examples	Ref.
Electrodes	Electrodes for lead–acid batteries	[67]
Fuel cells	Conductive titanium sub-oxide support materials in fuel cells	[73,74,75,76,77,78,79,80,81,82,83,84,85,86]
Remediation of aqueous waste and contaminated water	Electrocatalytic degradation for wastewater treatment	[87,88,89,90,91]
Ti_4_O_7_ reactive membranes	Membranes for advanced electrochemical oxidation processes	[92,93,94,95,96,97,98]
Batteries	As a sulfur host in Li_2_S battery, and conductive additive for improving performance of Li_2_S battery	[43,60,99,100,101,102,103,104,105]
Solar cells	The TiO/TiO_x_ layer can enhance the absorption of sunlight, thus increasing solar conversion efficiency	[106,107,108,109,110]
Sensors	Investigation of using nanostructured titanium sub-oxides as sensor materials for the determination of gaseous materials	[111,112,113,114,115]
Electronic and photonic materials	Nanostructured Ti_4_O_7_ in TiO_2_ resistive switching memory. Ti_3_O_5_ for light-triggered metal semiconductor transition. Titanium sub-oxides as thermoelectric materials	[3,7,16,116]
Biological applications	Coating material for medical devices	[117]

## Data Availability

Not applicable.
